# A Cross-Sectional Survey on Telemedicine Use for Doctor-Patient Communication

**DOI:** 10.7759/cureus.10402

**Published:** 2020-09-12

**Authors:** Aelia Akbar, Aqsa Iqbal, Dominic Gaziano, Filip Gasior, Ayesha J Zaidi, Anum Iqbal, Abigail Silva

**Affiliations:** 1 Public Health, Loyola University Medical Center, Chicago, USA; 2 Internal Medicine, Northeast Georgia Medical Center, Gainesville, USA; 3 Internal Medicine, Amita Health, Chicago, USA; 4 Internal Medicine, University of Illinois at Chicago, Chicago, USA; 5 Internal Medicine, Liaquat University of Medical and Health Sciences, Hyderabad, PAK

**Keywords:** telemedicine, patient-doctor communication, telehealth, patient portal, patient-doctor relation

## Abstract

Introduction

Use of computers for doctor-patient communication is increasing. Considering effective doctor-patient communication is important for good health outcomes. This study helps to determine the level of acceptance of telemedicine in general public and factors associated with it.

Methods:

This survey with cross-sectional analysis comprised a brief survey with 15 questions. The survey was distributed in public places to determine the opinions of the general public.

Results

Randomly selected 125 participants completed the questionnaire. Synchronous telemedicine was favored by young people (82% in the 18-34 age group vs 37.5% of participants aged >55 years; p<0.01), those with a higher education level (46.7% of non-college-educated persons vs 80.6% of college-educated persons; p<0.01), and frequent computer users (67% who used a computer for less than two hours a month vs 86.5% of those who used a computer more than hours a month; p=0.03). Asynchronous communication, like sending health information to doctors via a safe portal was acknowledged mostly by people who had used patient portals in the past (84.1% vs 65.4%; p=0.02). Use of patient portals was less among older users and senior citizens (20.8% use in the age group >55 vs. 51.3% in the age group 35-53 years vs. 71% in age group 18-34 years). Receiving video education for specific health concerns was favored by those who used a computer frequently (94.6% who used a computer more than two hours a month vs 77% who used a computer less than two hours a month; p =0.02).

Conclusion

Telemedicine is generally favored, but physicians should be mindful about older people as they may not feel comfortable. Step by step guidance should be provided especially to senior citizens for telemedicine and portal use.

## Introduction

Effective doctor-patient communication is important for history gathering, building trust, and patient compliance. Studies have shown that effective communication between patient and physician is associated with positive health outcomes [1-4]. Telemedicine is the exchange of medical information through electronic devices between patients and physicians [5,6]. Telemedicine may be used for asynchronous or synchronous doctor-patient communication [7]. In asynchronous communication, the doctor and patient are not connected at the same time. For example, communication through emails, safe patient portals, interpretation of diagnostic tests and pre-visit completion of surveys by patients. Synchronous communication refers to communication when the doctor and patient are connected at the same time, and is usually in the form of video conferencing [7].

Telemedicine increases access to care, saves travel time. and helps bring down costs. During the pandemic of COVID-19, telemedicine became an effective way to prevent the spread of infection without disrupting patients’ access to health care. Studies have shown increased access to healthcare and positive health outcomes when telemedicine was used for the management of chronic illnesses, like asthma in schools, and diabetes in rural areas [8,9].

The purpose of this study was to explore people’s perspectives regarding the use of telemedicine for patient access to healthcare.

## Materials and methods

Study design and population

Our survey study took place from 2017 to 2019 in general public places like parks, bus stops, and convenience stores in the city of Chicago, Illinois. People aged 18 years and older were eligible to participate. A written questionnaire was distributed among the people by anonymous study investigators and provided verbal consent to participate. Respondents to the survey remained anonymous and therefore the study was exempt from IRB approval. The individuals who completed the questionnaire did not receive any financial compensation. 

Questionnaire

A research questionnaire consisting of 14 questions was developed by the study investigators. We examined the general acceptance of doctor-patient online communication. We examined two scopes of doctor-patient interaction according to participants’ characteristics, (1) acceptance of synchronous telemedicine with doctor communicating directly via computer; and (2) acceptance to asynchronous telemedicine, i.e., receiving health information from doctors on cellphone or computer, receiving appointment reminders on a cellphone, sending health information to the doctor through a safe portal, some health questions being answered before visiting the doctor, receiving wellness reminders through a computer, receiving video education from a doctor, and the doctor seeing and evaluating their patient’s health information on an electronic health application.

Data collection and analysis

A brief verbal review of the study was provided to the people at public places by the study investigators. Participants were randomly selected. Those who verbally consented to participate were asked to fill out the anonymous paper survey questionnaire at the meeting place. The data was saved to an Excel file. Stata version 14 (StataCorp., College Station, Texas) was used to perform the statistical analysis. A descriptive analysis was obtained followed by bivariate analysis to detect a statistical association between the patient’s independent variables and their standpoint (Yes/No) on the acceptance of use of computer for communication with their doctor. A p-value of <0.05 was considered statistically significant. Chi-square test was used to compare the response of participants according to their characteristics. For data where the number of participants was less than seven, Fisher's exact test was performed. 

## Results

Study participants

There were 125 patients who completed the questionnaire. The mean age of participants was 38.4 (SD 15.1 [SD: standard deviation) years with ages ranging from 18 to 81 years. Approximately half of the participants were below the age of 35 years, 31.2% were 35-54 years and 19.2% were 55 years and older. Out of the total number of participants, almost two-thirds (75.6%) had attended college (Table 1).

**Table 1:** Characteristics of 125 participants who completed the survey questionnaire.

Characteristics	n (%)
Age (%)	
18-34 year	62 (49.6)
35-54 year	39 (31.2)
>55 year	24 (19.2)
Education level (%)	
Did not attend college	30 (24.4)
Attended College	93 (75.6)
Use computer (%)	109 (87.2)
Use cell phone	121 (97.6)
Use computer to try to diagnose your self	77 (61.6)
Use computer to research medical condition	99 (79.8)
Hours of use of computer in a month for your health	
< 2 hr	85 (69.7)
>2 hrs	37 (30.2)
Have used a patient portal before	69 (55.2)

Computer use pattern among study participants:

The majority of participants who completed the survey used computer (87.2%) and cellphones (97.6%) in their everyday life. The percentage of participants who used computers to diagnose themselves was 61.6%, and the percentage of those who used computers to research medical conditions was 79.8%. Majority of participants 69.7% (85/125) used a computer for less than two hours a month. Almost half (55.2%) of participants had used a patient portal in the past (Table 1).

Opinions about using a computer for doctor-patient communication

The idea that it is useful when the doctor uses a computer to communicate directly with the patient / synchronous communication was shared by 72% of participants. The majority (81.1%) were in favor of receiving the health information from their doctor on their cellphone and computer. The majority were in favor to receive appointment reminders (88.7%) on their cellphones and receiving wellness reminders on their computer (82.1%). The majority were comfortable in sharing their health information with their doctors through a safe portal (75.8%) and favored the idea that some of their health questions could be answered before their visit to the doctor (79.8%). Participants also liked the idea to receive video education regarding their specific health concerns (82.9%), and the use of health apps by the doctor to see and evaluate their health information (78.9%) (Table 2).

**Table 2:** Participants responses to survey questions

Synchronous Doctor-Patient Communication	Number	Percent
Would you find it useful if your doctor communicated with you directly through the computer?		
Yes	90	72
No	35	28
Asynchronous Doctor-Patient Communication		
Open to receive helpful health information from doctor on cellphone or computer		
Yes	99	81.1
No	23	18.9
Open to receive appointment reminders on cellphone		
Yes	110	88.7
No	14	11.3
Would you be comfortable sending your health information to a doctor through a safe portal		
Yes	94	75.8
No	30	24.2
Would you like some of your health questions answered before your visit to the doctor		
Yes	99	79.8
No	25	20.2
Open to receiving wellness reminders from your doctor through computer		
Yes	101	82.1
No	22	17.9
Would it be helpful if your doctor send you video education regarding your specific health concerns		
Yes	102	82.9
No	21	17.1
Do you think it would be helpful if your doctor could see and professionally evaluate the information on a health app that you are using?		
Yes	97	78.9
No	26	21.1

Influence of patient characteristics and use of computer on virtual patient-physician communication

Synchronous Communication

**Figure 1:**
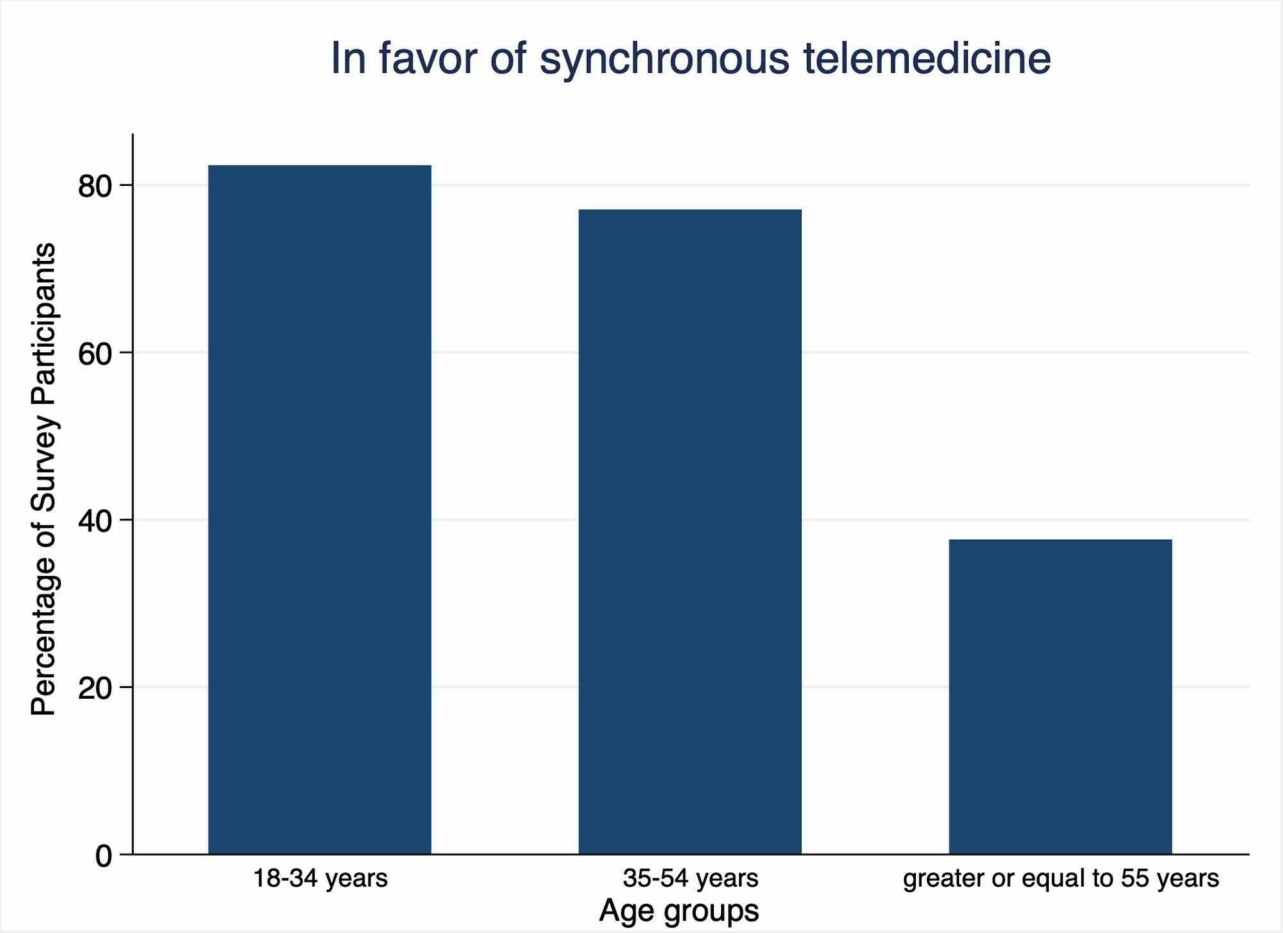
Percentage of survey participants in favor of synchronous communication according to age groups.

The patients’ characteristics and computer usage affected their opinions towards virtual patient-physician communication (as shown in Table 3). Younger patients and those with higher education level were in favor of the doctor communicating with them directly on the computer as compared to older patients and patients with lower education level, respectively 82% (in the age range of 18-34 vs 37.5% aged 55 years and older; p <0.01) and 46.7% (not college-educated vs 80.6% of those who attended college). Figure 1 shows the percentage of participants who were in favor of synchronous doctor-patient communication according to age. Hours of computer use also affected this opinion (67% who used the computer for less than two hours a month vs 86.5% of those who used the computer more than hours a month; p <0.05) with those who used a computer for more than two hours per day preferred synchronous communication (Table 3). The use of a patient portal did not affect the opinion for the synchronous communication (p>0.05).

**Table 3:** Participants’ responses to survey questions for synchronous doctor-patient communication according to participant characteristics:

	Yes, n (%)	No, n (%)	p-value
Age Groups (years)			<0.001
18-34	51 (82.3)	11 (17.7)	
35-54	30 (76.9)	9 (23.2)	
≥55	9 (37.5)	15 (62.5)	
Education			<0.001
Did Not Attend College	14 (46.7)	16 (53.3)	
Attended College	75 (80.6)	18 (19.4)	
Hours of Computer Use			0.03
< 2 hours	57 (67.1)	28 (32.9)	
> 2 hours	32 (86.5)	5 (13.5)	
Used Patient Portal Before			0.08
Yes	54 (78.3)	15 (21.7)	
No	36 (64.3)	20 (35.7)	

Asynchronous Communication

**Figure 2:**
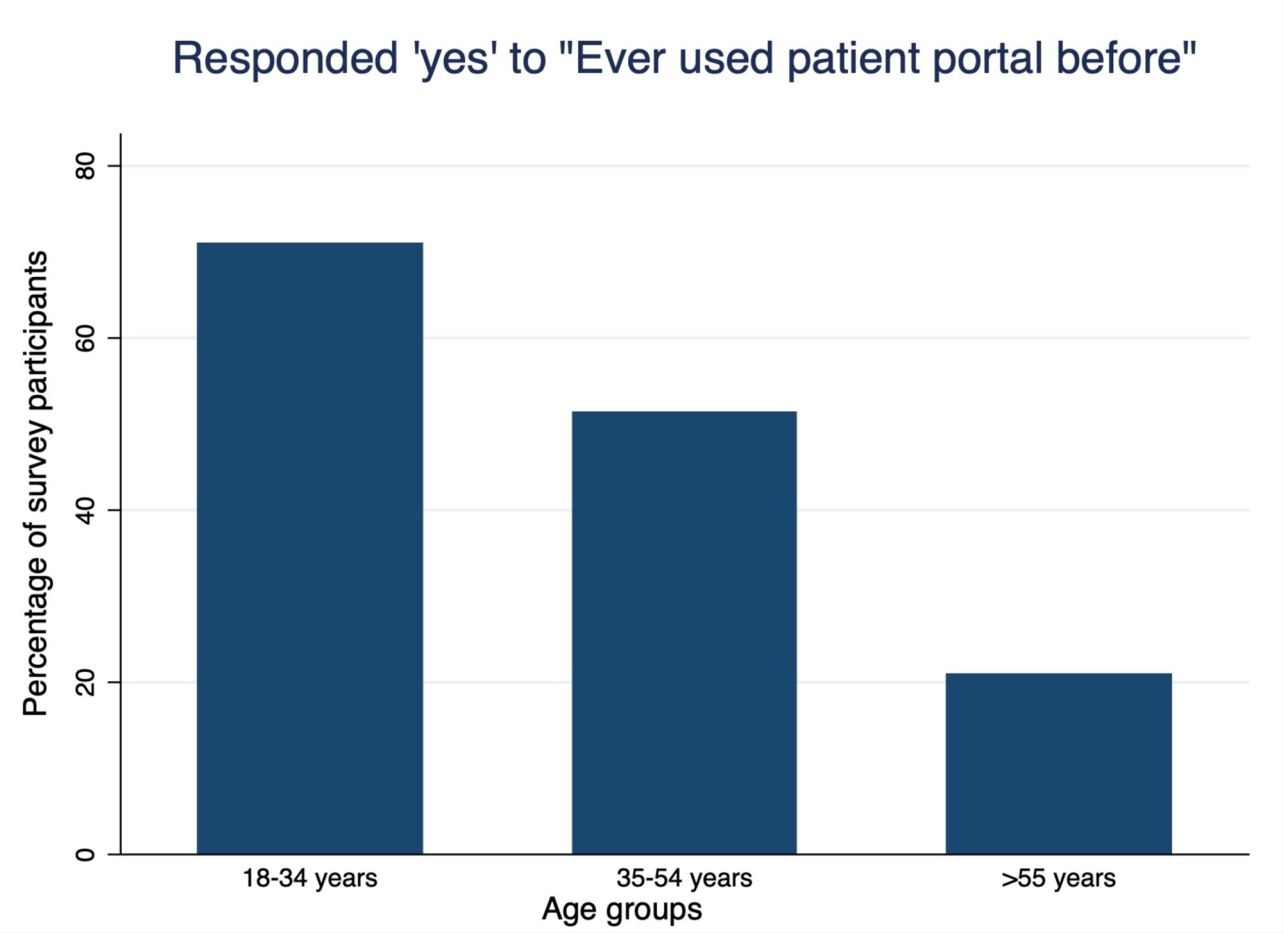
Percentage of survey participants who responded ‘yes’ to “have you ever used patient portal before” according to age groups.

**Figure 3:**
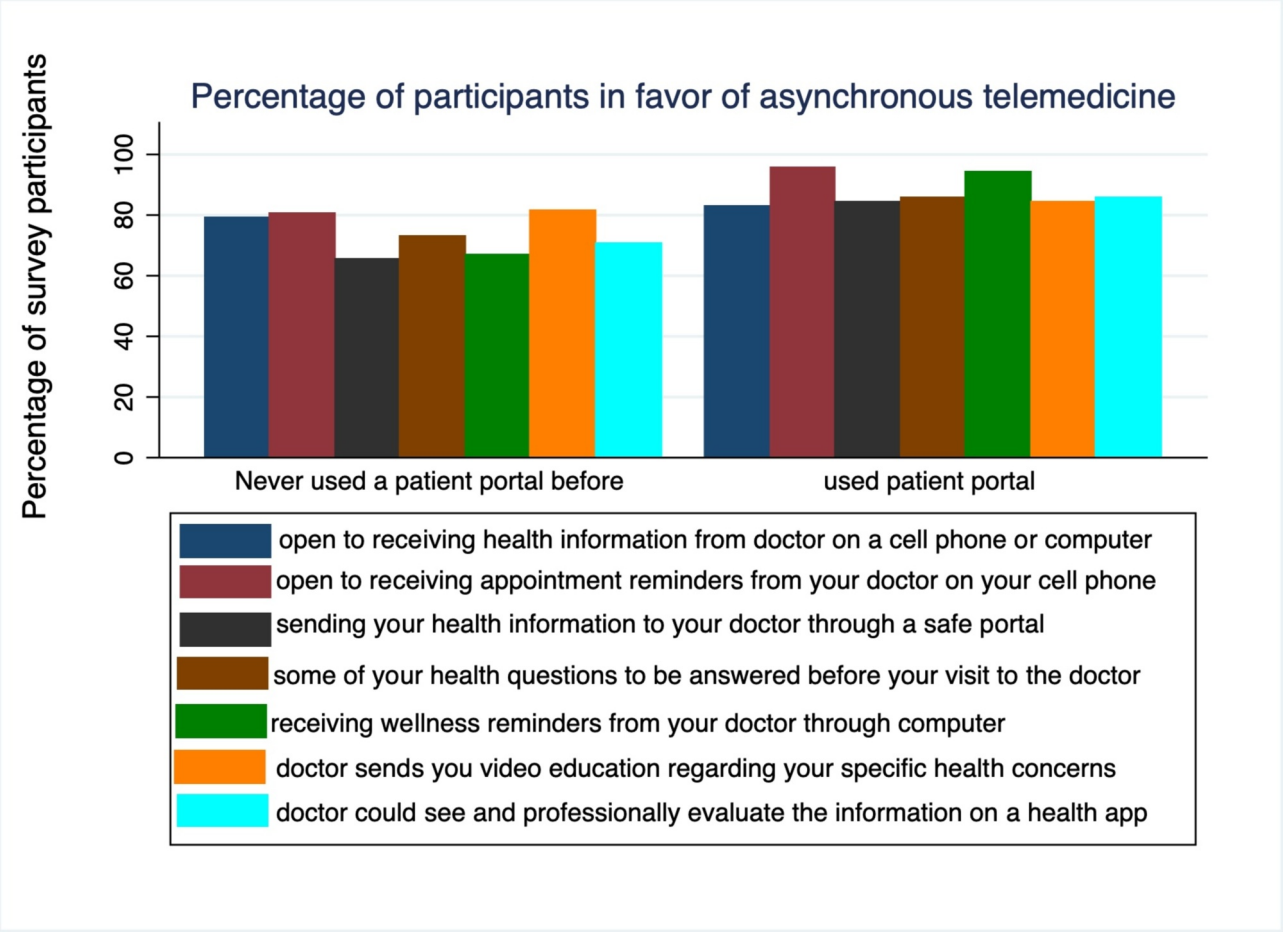
Percentage of survey participants who responded ‘yes’ to asynchronous telemedicine questions according to prior use of patient portal. Q1: Would you be open to receiving health information from your doctor on a cellphone or computer? Q2: Would you be open to receiving appointment reminders from your doctor on your cellphone? Q3: Would you be comfortable sending your health information to your doctor through a safe portal? Q4: Would you like some of your health questions be answered before your visit to the doctor? Q5: Would you be open to receiving wellness reminders from your doctor through computer? Q6: Do you think it would be helpful if your doctor send you video education regarding your specific health concerns? Q7: Do you think it would be helpful if your doctor could see and professionally evaluate the information on a health app?

Figure 2 shows the use of patient portal according to age groups, and older age people were less likely to have used portal in the past (20.8% use in age group 55 and older vs. 51.3% in those aged 35-53 years vs 71% in the 18-34 age group). Figure 3 shows the responses to asynchronous telemedicine according to prior use of patient portal in general public with mostly (>60%) in favor of using it. Table 4 shows the responses of participants to questions on asynchronous communication according to age, education level, computer usage and prior use of patient portal.

**Table 4:** Participants’ responses to survey questionnaire for asynchronous doctor-patient communication according to participant characteristics. * The total number of responses may not add to 125 in all questions because of missing data in some responses.

Survey Questions	Yes, n (%)	No, n (%)	p-value
Q:1 Would you be open to receiving helpful health information from your doctor on a cell phone or computer?			
Age groups (years)			0.90
18-34	49 (81.7)	11 (18.3)	
35-54	30 (78.9)	8 (21.0)	
≥55	20 (83.3)	4 (16.7)	
Education			0.35
Not attended college	22 (75.9)	7 (24.1)	
Attended college	76 (83.5)	15 (16.5)	
Hours of computer use			0.28
< 2 hours	64 (78.0)	18 (21.9)	
> 2 hours	32 (86.5)	5 (13.5)	
Used patient portal before			0.64
Used portal before	57 (82.6)	12 (17.4)	
Not used portal before	42 (79.2)	11 (20.7)	
Q:2 Would you be open to receiving appointment reminders from your doctor on your cell phone?			
Age groups (years)			0.23
18-34	58 (93.5)	4 (6.4)	
35-54	32 (84.2)	6 (15.8)	
≥55	20 (83.3)	4 (16.7)	
Education			0.89
Not attended college	27 (90.0)	3 (10.0)	
Attended college	82 (89.1)	10 (10.9)	
Hours of computer use			0.05
< 2 hours	72 (84.7)	13 (15.3)	
> 2 hours	35 (97.2)	1 (2.8)	
Used patient portal before			0.008
Used portal before	65 (95.6)	3 (4.4)	
Not used portal before	45 (80.4)	11 (19.6)	
Q:3 Would you be comfortable sending your health information to doctor through a safe portal?			
Age groups (years)			0.081
18-34	49 (79.0)	13 (20.9)	
35-54	31 (81.6)	7 (18.4)	
≥55	14 (58.3)	10 (41.7)	
Education			0.08
Not attended college	19 (63.3)	11 (36.7)	
Attended college	73 (79.3)	19 (20.6)	
Hours of computer use			0.79
< 2 hours	64 (76.2)	20 (23.8)	
> 2 hours	29 (78.4)	8 (21.6)	
Used patient portal before			0.02
Used portal before	58 (84.1)	11 (15.9)	
Not used portal before	36 (65.4)	19 (34.5)	
Q:4 Would you like some of your health questions answered before your visit to the doctor?			
Age groups (years)			0.47
18-34	50 (81.9)	11 (18.0)	
35-54	32 (82.0)	7 (17.9)	
≥55	17 (70.8)	7 (29.2)	
Education			0.49
Not attended college	22 (75.9)	7 (24.1)	
Attended college	76 (81.7)	17 (18.3)	
Hours of computer use			0.51
< 2 hours	66 (78.6)	18 (21.4)	
> 2 hours	31 (83.8)	6 (16.2)	
Used patient portal before			0.08
Used portal before	59 (85.5)	10 (14.5)	
Not used portal before	40 (72.7)	15 (27.3)	
Q:5 Would you be open to receiving wellness reminders from your doctor though computer?			
Age groups (years)			<0.001
18-34	56 (91.8)	5 (8.2)	
35-54	34 (87.2)	5 (12.8)	
≥55	11 (47.8)	12 (52.2)	
Education			<0.001
Not attended college	16 (55.2)	13 (44.8)	
Attended college	84 (91.3)	8 (8.7)	
Hours of computer use			0.54
< 2 hours	68 (81.9)	15 (18.1)	
> 2 hours	32 (86.5)	5 (13.5)	
Used patient portal before			<0.001
Used portal before	65 (94.2)	4 (5.8)	
Not used portal before	36 (66.7)	18 (33.3)	
Q:6 Do you think it would be helpful if the doctor sends you video education regarding your specific health concern?			
Age groups (years)			0.07
18-34	50 (81.9)	11 (18.0)	
35-54	36 (92.3)	3 (7.7)	
≥55	16 (69.6)	7 (30.4)	
Education			0.07
Not attended college	20 (71.4)	8 (28.6)	
Attended college	80 (86.0)	13 (13.9)	
Hours of computer use			0.02
< 2 hours	64 (77.1)	19 (22.9)	
> 2 hours	35 (94.6)	2 (5.4)	
Used patient portal before			0.71
Used portal before	58 (84.1)	11 (15.9)	
Not used portal before	44 (81.5)	10 (18.5)	
Q:7 Do you think it would be helpful if your doctor could see and professionally evaluate the information on a health app that you are using?			
Age groups (years)			0.06
18-34	51 (83.6)	10 (16.4)	
35-54	32 (82.0)	7 (17.9)	
≥55	14 (60.9)	9 (39.1)	
Education			0.04
Not attended college	18 (64.3)	10 (35.7)	
Attended college	77 (82.8)	16 (17.2)	
Hours of computer use			0.49
< 2 hours	65 (78.3)	18 (21.7)	
> 2 hours	31 (83.8)	6 (16.2)	
Used patient portal before			0.04
Used portal before	59 (85.5)	10 (14.5)	
Not used portal before	38 (70.4)	16 (29.6)	

The participant age, education level, and use of computer and patient portal did not affect their opinion about receiving helpful health information from a doctor via computer, and the majority were in favor of it. 

The idea of receiving appointment reminders on cell-phone was mostly favored by those who used patient portal with statistical significance, but it was not affected by participants age, usage of computer or education level. Only three participants out of total 125 did not use cell phones so we did not analyze the opinion about receiving appointment reminders on cell phone according to cellphone usage. 

Those who had not used a patient portal in the past were less likely to be comfortable sending health information to their doctors via a safe portal as compared to those who had used it in the past (65.4% vs 84.1%; p=0.02). Age, education level and frequency of computer use did not affect their attitudes about sending information through a safe portal.

Patient characteristics, computer use and prior use of patient portal did not affect their opinion about health questions being answered prior to their visit. 

The idea of receiving wellness reminders through a computer was favored by younger patients (91.8% of 18-34 years of age vs 47.8% of 55 years of age; p<0.01), with higher education level (91.3% who attended college vs 55.2% who did not attend college; p<0.01), and those who had used patient portals in the past (94% vs 66.7%; p <0.01), but infrequent use of computers did not affect the idea of receiving wellness reminders through a computer (p >0.05). 

The idea of receiving video education for specific health concerns was particularly favored by those who used computer frequently (94.6% who used computer >2 hours vs 77% who used computer <2 hours a month; p =0.02). Age, education level and portal use did not affect this opinion. 

The opinion about use of health app by doctors to professionally evaluate health conditions did not differ by patient characteristics, frequency of computer use or use of patient portal, but it differed by education level. 

Figure 2 shows the use of patient portal according to age groups, and older age people were less likely to have used portal in the past (20.8% use in respondents aged 55 and older vs. 51.3% in the 35-53 year age group vs 71% in those aged 18-34 years ). Figure 3 shows the responses to asynchronous telemedicine according to prior use of patient portals in general public with mostly (>60%) in favor of using it. 

## Discussion

Computers have become an integral part in clinical practice without detrimental effects on patient satisfaction [10]. Good patient-physician communication is important for healing [11]. In this evolving era of medicine and communication, the purpose of our study was to inquire about the general public’s perspectives about the use of telemedicine as well as suggest some methods for improvement. We found that majority of people were comfortable with their doctors communicating with them directly via computer, also known as synchronous telemedicine, a finding common to prior studies [12-14]. However, previous studies did not analyze these opinions according to patient characteristics. In our study, younger age, higher educational attainment and computer usage were identified as strong indicators for acceptance of synchronous telemedicine.

We evaluated general acceptance by participants to asynchronous telemedicine and provided some suggestions; using a patient portal, health concerns answered before a visit to doctor’s clinic, receiving wellness reminders through the computer, receiving educational videos regarding specific health concerns, and use of health app by physicians for health evaluation. However, lack of prior use of a patient portal hampered the acceptance of this way of communication (Figure 3). Patient portals allow patients to remain engaged in their healthcare decisions and easily communicate with their physicians. Our study showed less use of patient portals by older adults (Figure 2). The main barriers identified are lack of technology, health literacy and distrust of online devices by older patients [15-16]. Therefore, step by step guidance for the use of patient portals in this age group is necessary. 

We conducted the survey in general public and not just in physicians' clinics as there are many people who do not go to healthcare providers because of various reasons, such as lack of time and transportation. By conducting the survey in general public spaces, we were able to capture opinions of all types of people. Another strength of this study is that we categorized the people’s opinions according to age, education level, computer usage, prior use of patient portals, and evaluated their views about both asynchronous and synchronous telemedicine. However, our study was conducted before the onset of COVID-19 pandemic during which telemedicine was the only way for most people to get their healthcare needs. It is likely to get more positive responses if the study is conducted during or after the pandemic and this opens door to future studies. Our survey methods had several limitations as it was not specified for a particular group, and people with certain health problems could have answered differently as compared to those without any health problems. This opens doors to future studies to evaluate the acceptance of telemedicine on a case by case basis. For example, tele-neurology is widely accepted for mental health evaluation, refills of medicines, neurorehabilitation, outpatient consultation, as well as pediatric neurology [17].

## Conclusions

In conclusion, the use of computers for communicating with primary care physicians is generally accepted, however, clinicians should be mindful that older adults may not feel comfortable with synchronous telemedicine. People, especially older adults, should be encouraged to use computers and patient portals.

## Appendices

Online Wellness Survey

Q1: What is your age?

Q2: Do you use a computer at all? 

o   Yes 

o   No

Q2: Do you use a cellphone? 

o   Yes 

o   No

Q3: Would you be open to receiving helpful health information from your doctor on a cellphone or computer? 

o   Yes 

o   No

Q4: What is your education level at this point?

o   Not attained high school diploma

o   Graduate high school/ GED

o   Some college 

o   Graduate college 

o   Graduate degree

Q5: Would you be open to receiving appointment reminders from your doctor on your cellphone?

o   Yes 

o   No

Q6: Do you use a computer to try to diagnose yourself?

o   Yes 

o   No

Q7: Do you use a computer to research a medical condition?

o   Yes 

o   No

Q8: How many hours do you use a computer for your health information in a month?

o   Less than 1 hour

o   1 to 2 hours 

o   2 to 4 hours

o   4 to 8 hours 

o   More than 8 hours 

Q9: Have you used a patient portal before? (patient portals provide web access for your medical records from the hospital or doctor’s office)

o   Yes 

o   No

Q10: Would you find it useful if your doctor communicated with you directly on a computer?

o   Yes 

o   No

Q11:  Would you be comfortable sending your health information to your doctor through a safe portal?

o   Yes 

o   No

Q12: Would you like some of your health questions to be answered before your visit to the doctor?

o   Yes 

o   No

Q13: Would you be open to receiving wellness reminders from your doctor through the computer?

o   Yes 

o   No

Q14: Do you think it would be helpful if your doctor sends you video education regarding your specific health concerns?

o   Yes 

o   No

Q15: Do you think it would be helpful if your doctor could see and professionally evaluate the information on a health app that you are using?

o   Yes 

o   No
